# The *in Vitro* Biological Activity of the Brazilian Brown Seaweed *Dictyota mertensii* against *Leishmania amazonensis*

**DOI:** 10.3390/molecules190914052

**Published:** 2014-09-09

**Authors:** Amanda Silva dos Santos Aliança, Keicyanne Fernanda Lessa dos Anjos, Thiago Nogueira de Vasconcelos Reis, Taciana Mirely Maciel Higino, Maria Carolina Accioly Brelaz-de-Castro, Éverson Miguel Bianco, Regina Celia Bressan Queiroz de Figueiredo

**Affiliations:** 1 Departamento de Microbiologia, Centro de Pesquisa Aggeu Magalhães (CPqAM-FIOCRUZ), Av. Moraes Rego s/n Cidade Universitária, Campus da UFPE, Recife 50670-420, Brazil; E-Mails: Amanda_alianca@yahoo.com.br (A.S.S.A.); keicyanne1@yahoo.com.br (K.F.L.A.); taciana@cpqam.fiocruz.br (T.M.M.H.); 2 Departamento de Oceanografia, Universidade Federal de Pernambuco (UFPE), Recife 50740-550, Brazil; E-Mail: reistnv@gmail.com; 3 Departamento de Imunologia Centro de Pesquisa Aggeu Magalhães (CPqAM-FIOCRUZ), Av. Moraes Rego s/n Cidade Universitária, Campus da UFPE, Recife 50670-420, Brazil; E-Mail: carolina.brelaz@gmail.com; 4 Programa de Pós-graduação em Química, Fundação Universidade Regional de Blumenau (FURB), Campus 1, Rua Antonio da Veiga, 140, Blumenal 89012-900, Brazil; E-Mail: ebianco@chemist.com

**Keywords:** *Dictyota mertensii*, chemotherapy, *Leishmania amazonensi*, ultrastructure

## Abstract

Seaweeds present a wide variety of interesting bioactive molecules. In the present work we evaluated the biological activity of the dichloromethane/methanol (2:1) extract (DME) from the brown seaweed *Dictyota mertensii* against *Leishmania amazonensis* and its cytotoxic potential on mammalian cells. The extract showed significant inhibitory effect on the growth of promastigote forms (IC_50_ = 71.60 μg/mL) and low toxicity against mammalian cells (CC_50_ = 233.10 μg/mL). The DME was also efficient in inhibiting the infection in macrophages, with CC_50_ of 81.4 μg/mL and significantly decreased the survival of amastigote forms within these cells. The selectivity index showed that DME was more toxic to both promastigote (SI = 3.25) and amastigote (SI = 2.86) forms than to macrophages. Increased NO production was observed in treated macrophages suggesting that besides acting directly on the parasites, the DME also shows an immunomodulatory effect on macrophages. Drastic ultrastructural alterations consistent with loss of viability and cell death were observed in treated parasites. Confocal microscopy and cytometry analyzes showed no significant impairment of plasma membrane integrity, whereas an intense depolarization of mitochondrial membrane could be observed by using propidium iodide and rhodamine 123 staining, respectively. The low toxicity to mammalian cells and the effective activity against promastigotes and amastigotes, point to the use of DME as a promising agent for the treatment of cutaneous leishmaniasis.

## 1. Introduction

The metabolic and physiologic ability of marine organisms, especially seaweeds, in dealing with highly complex and hostile environments have resulted in the evolution of a number of secondary metabolic pathways which have high prospects of yielding natural products with unique chemical structures. In this regard, different natural products isolated from marine organisms, mainly from invertebrates and seaweeds, have been reported to possess a broad spectrum of pharmacological properties, such as antiviral [1,2,3], antiprotozoal [4,5,6,7,8], antibacterial [9], antioxidant [10], antifungal [11], cytotoxic [12,13] and antitumoral activities [14,15]. In seaweeds, these biological activities have been attributed to the presence of diverse secondary metabolites such as terpenes, acetogenins and polyphenols, including many unusual halogenated compounds [16,17]. 

The success in the area of bioactive marine natural products has been already proven by the increasing number of new compounds in pre- or clinical evaluation, which are of interest from the point of view of potential drug development [18,19].

Brazil is a continental country; with 8500 km of Atlantic coastline that supports an exclusive and rich diversity of endemic marine fauna and flora that remains an open field of investigation in the search for novel bioactive secondary metabolites with potential medicinal properties. However, so far only a few classes of Brazilian marine organisms have been investigated for their chemical and pharmacological properties. In this regard the identification of Brazilian organisms with significant biotechnological potential for the development of new chemotherapeutic agents, particularly those for treatment of neglected parasitic diseases, is an important goal to be achieved [20].

Leishmaniasis is a group of zoonotic tropical diseases caused by protozoan parasites of the genus *Leishmania*. These diseases are present in 88 countries, with about 12 million people infected and 350 million at risk of infection. It is estimated that about 2 million new cases occur worldwide each year, with 500,000 cases of visceral leishmaniasis and 1,500,000 of the cutaneous form of the disease [21,22]. 

The disease in humans presents two most common clinical forms: visceral (VL) and cutaneous (CL) with a broad range of clinical manifestations depending on both the parasite species and the host immune response. Some forms of cutaneous leishmaniasis, mainly the diffuse and mucosal leishmaniasis, can be considered severely disfiguring and extremely debilitating, leading to serious consequences on the psychological profile of the affected individuals [23]. 

The pentavalent antimonials, sodium stibugluconate (Pentostan^®^) and meglumine antimoniate (Glucantime^®^) are still the first line drugs for the treatment of all clinical forms of leishmaniasis. However, both drugs cause several side effects and resistance of parasite to the treatment has been also reported. Pentamidine and amphotericin B have been used as second-line alternative treatments. However, these drugs also present side effects and require parenteral administration [24]. 

The brown seaweeds of the family *Dictyotaceae* are recognized as a rich source of monocyclic, bicyclic and tryciclic diterpenes as major secondary metabolites [25,26,27,28]. Many of these chemical molecules have been reported as antiprotozoal agents. Previous studies by Dos Santos *et al.* [6] and Bianco *et al.* [20] have demonstrated the trypanocidal and leishmanicidal potential of invertebrates and seaweeds collected along the Brazilian coast, including those belong to the genus *Dictyota*. However, to our knowledge, there are no reports in the literature on the antiprotozoal activity of the species *Dictyota mertensii*. In this regard, the biological activity of the extract obtained from this seaweed, collected from the Northastern Brazilian coast, was evaluated against *Leishmania amazonensis*. Our results indicate that the leishmanicidal activity of DME is associated with mitochondrial depolarization, which leads to the loss of parasite viability and cell death independent of necrosis.

## 2. Results and Discussion

The genus *Dictyota* Lamouroux 1809 is one of the most prolific in the production of secondary metabolites such as, fat acids, sterols and terpenes, of which over 300 different compounds were reported [27]. Therefore, these seaweeds have high potential to yield new and structurally unique natural products with biological activity against cutaneous leishmaniasis.

**Figure 1:**
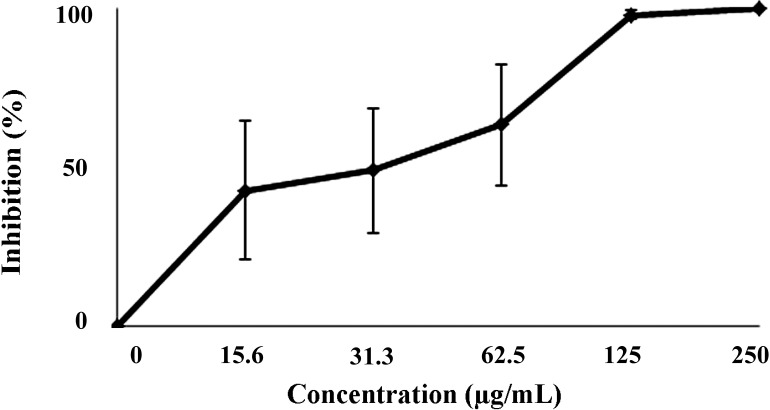
Effects of DME on the growth of promastigotes forms of *Leishmania amazonensis*.

In the present study the crude extract of *D. mertensii* obtained from a dichloromethane/methanol solvent mixture (2:1) (DME) was tested for its leishmanicidal and cytotoxic activities. Our results showed that DME presented a dose-dependent effect on the promastigote growth, reaching 100% growth inhibition at 250 μg/mL of DME (Figure 1). The estimated IC_50_/72 h (the concentration of DME that inhibited by 50% the promastigotes growth) was 71.60 ± 9.29 µg/mL.

The data points represent the median of three experiments ± SD. Each set of experiments was repeated twice.

An important criterion in the search of new compounds with leishmanicidal activity is their low cytotoxicity to mammalian cells [29]. In this regard, the cytotoxic potential of DME on macrophages was assessed by the MTT assay. The DME presented low toxicity towards macrophages with a CC_50_ (50% cytotoxic concentration) value of 233.10 ± 25.5 µg/mL. The toxicity of DME to macrophages and its activity against promastigotes were compared to determine the selectivity index (SI = CC_50_ macrophages/IC_50_ promastigote). Our results showed that DME was about three times more toxic to the promastigotes than to mammalian cells (SI = 3.25).

**Figure 2:**
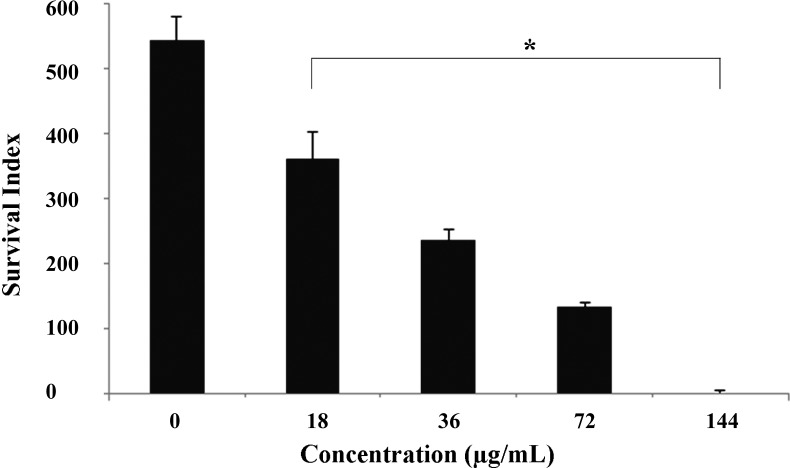
Survival index of *Leishmania amazonensis* within macrophages after 18 h of treatment with the DME.

**Figure 3:**
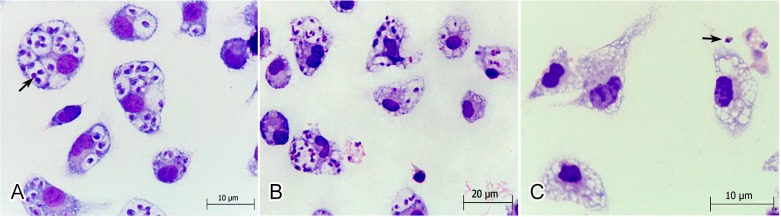
Light microscopy of the effects of DME on *L. amazonensis*-infected Balb/c peritoneal macrophages. (**A**) Giemsa-stained macrophages infected with amastigote (arrow); (**B**) Giemsa-stained macrophages infected with amastigotes and treated with the 72 µg/mL and (**C**) Giemsa-stained macrophage infected with amastigotes and treated with the 144 µg/mL DME. Note in (C) the absence of intracellular amastigotes.

Because the intracellular amastigotes are responsible for the clinical manifestations of leishmaniasis [30], the effects of DME on this relevant life stage of parasite was also evaluated. Our data showed that DME significantly inhibited (*p* < 0.05) the survival of amastigote forms within the peritoneal macrophages, at all tested concentrations tested, as compared with the non-treated cells (Figure 2), with an IC_50_ value after 18 h of drug incubation of 81.4 ± 1.7 µg/mL and a SI value of 2.86. At a higher concentration (144 µg/mL), DME completely inhibited the infection of macrophages, as observed in the Giemsa-stained samples (Figure 3). Although extracellular promastigotes seem to be slightly more susceptible than intracellular amastigotes, it is important to emphasize that the time of the drug incubation used for this latter life stage was considerably shorter than those used for promastigotes, so it is possible that at longer times of exposure to DME, the activity of this extract against amastigotes may be higher than those found for promastigote forms.

The data points represent the median of three experiments ± SD. Each set of experiments was repeated twice. * Significant values (*p* < 0.05).

**Figure 4:**
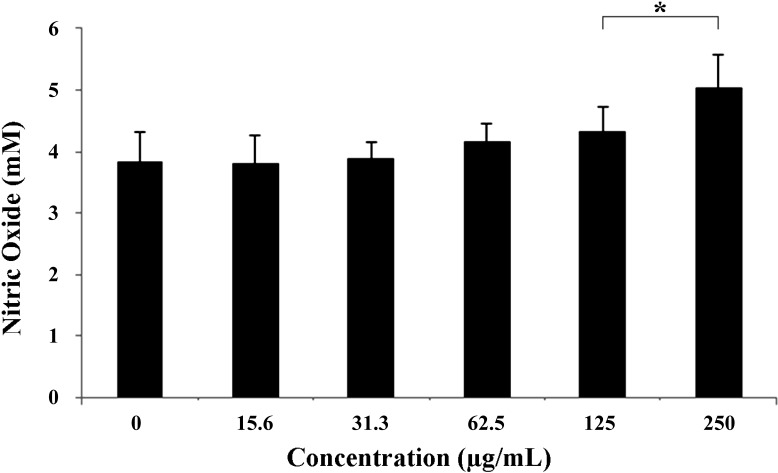
Effect of the DME on the production of nitric oxide (µM) by macrophages.

The observed effects of DME against the amastigotes prompted us to investigate whether the DME acts directly on the parasite or indirectly by improving the microbicidal function of macrophages. Our results showed that DME induced an enhancement of NO production by macrophages (*p* < 0.05) at 125 and 250 μM DME (Figure 4), suggesting that besides acting directly on the parasites, DME also had an immunomodulatory effect on macrophages increasing their microbicidal activity. Nitric oxide has been demonstrated to be an important effector molecule responsible for mediating intracellular killing of leishmania parasites [31]. However, these parasites have developed highly sophisticated mechanisms to inhibit the synthesis of NO directly or indirectly by blocking the IL12 synthesis and consequently, the development of Th1 cells response [30]. The reestablishment of NO production by macrophages at higher concentrations of DME may lead to a complete abolishment of macrophage infection as observed in Figure 3C.

* Significant difference compared to the control cells (*p <* 0.05), ANOVA; Dunnett test.

**Figure 5:**
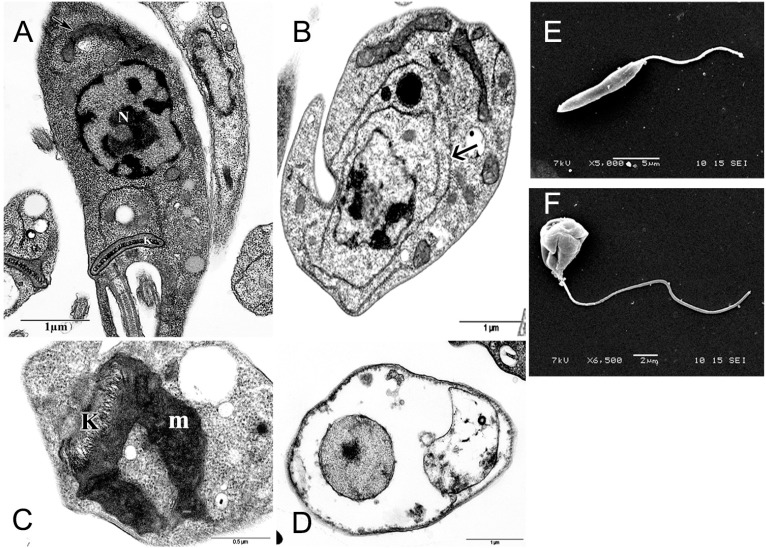
Effect of DME on the parasite ultrastructure as observed by transmission (**A**–**D**) and scanning (**E**,**F**) electron microscopy; (A) A control promastigote form showing a morphologically preserved mitochondrion (arrow), nucleus (N) and kinetoplast (K); (B) Promastigote forms treated with IC_50_ DME. Note the presence of endoplasmic reticulum profiles (arrow) surrounding the organelles; (C) High magnification of mitochondrion (m) of a promastigote treated with IC_50_ DME; (D) Drastically damaged promastigote treated with 2 × IC_50_ DME; (E) Scanning electron microscopy of a control and (F) 2 × IC_50_ DME-treated promastigote form.

In order to identify putative intracellular targets of the DME an ultrastructural analysis of treated and control promastigotes was performed. As observed by transmission electron microscopy, untreated parasites are characterized by elongated body, a nucleus with an evidenced central nucleolus and chromatin associated with the inner sheet of the nuclear envelope. A single mitochondrion containing a region of concentrated DNA denominated kinetoplast was observed (Figure 5A). Cells treated with IC_50_ DME presented as main characteristics an enhancement of endoplasmic reticulum profiles, suggestive of autophagy and loss of nuclear organization, characteristic of apoptosis (Figure 5B). A swollen mitochondrion, presenting disorganization of mitochondrial cristae and k-DNA could be detected in most treated cells at the both concentrations tested (Figure 5C). A complete loss of cytoplasmic content and deterioration of organelles, which are compatible with loss of the parasite membrane integrity and cell death, could be observed mainly in cells treated with 2 × IC_50_ DME (Figure 5D). Scanning electron microscopy assay (Figure 5E,F) showed a characteristic control cell presenting an elongated cell body and a smooth membrane (Figure 5F). Cells treated with 2 × IC_50_ showed round shape, swelling cell body and winkled membrane (Figure 5E).

Trypanosomatids, including the different species of genus *Leishmania* have a single mitochondrion exhibiting unique and structural and functional characteristics that are remarkably distinct from host mammalian cells [32,33]. These features make this organelle an important target for chemotherapeutic drugs. Treatment of the parasites with DME induced severe damage in the parasite mitochondrion. Alterations in the trypanosomatids’mitochondria have been reported in the literature in response to a number of drugs, showing the high susceptibility of this organelle to chemotherapeutic agents [34,35,36].

Because our ultrastructural analysis pointed towards the parasite mitochondrion as a putative intracellular target for DME action, we evaluated the mitochondrial membrane potential by using the fluorescent probe rhodamine 123. In eukaryotic cells, the active pumping of H^+^ during the electron transfer along the respiratory chain maintains the electrochemical potential of mitochondria and supports the integrity and function of this organelle. The imbalance of mitochondrial membrane potential could lead to a decrease of ATP, reduction in the transcription and translation of mitochondrial genes and increase in the ROS production, leading to cell death by apoptosis or necrosis [37,38,39]. Contrary to the mammalian cells kinetoplastids parasites have a single mitochondrion and rudimentary antioxidant defenses, as well as a set of alternative oxidases (AOX) not present in the host cells. By using metabolic ways different from those of host parasites can easily adapt to new environment conditions, which is crucial to their survival [33]. These parasite peculiarities can explain the high susceptibility of parasite mitochondrion to DME treatment and the selectivity of this extract towards the parasites as demonstrated in the present study.

**Figure 6:**
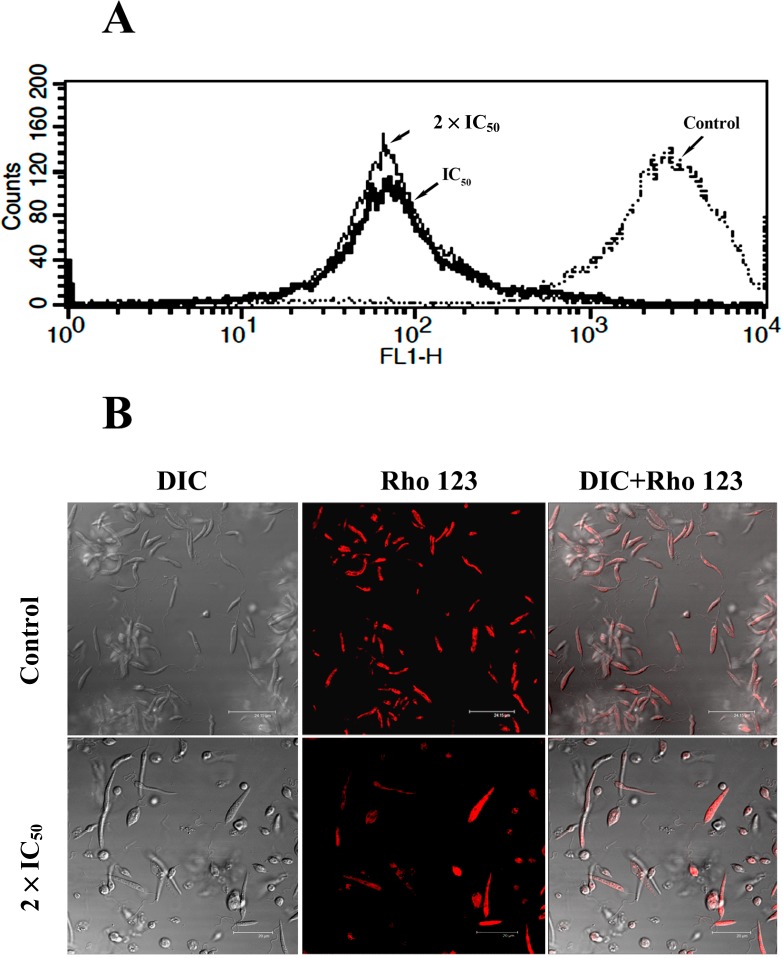
The Effects of DME on the mitochondrial membrane potential of *L. amazonensis* promastigotes. (**A**) Flow cytometry overlay histograms of the controls and DME-treated cells and stained with Rho 123. (**B**) Confocal image of the control and treated-cells. All control cells presented intense Rho123 labeling and preserved morphology. Cells treated with 2 × IC_50_ displaying a vanished fluorescence and drastic morphological changes.

Our flow cytometry assays showed a marked decrease in the Rho 123 fluorescence signal in both the IC_50_ and 2 × IC_50_-treated cells compared to the control cells (Figure 6A) The calculated IV values were −0.95 (IC_50_) and −0.97 (2 × IC_50_). The confocal images corroborate the data from flow cytometry, showing an intense Rho 123 labeling in the control cells indicative of a strong mitochondrial activity. Conversely, only a vanished and diffuse red fluorescence signal, indicative of strong mitochondrial depolarization, could be observed in the cells treated with 2 × IC_50_ of DME (Figure 6B).

Previous studies on the leishmanicidal activity of the sesquiterpene elatol, isolated from the red seaweed *L. dendroidea* [5] and the diterpene 4-Acetoxydelastane isolated from the brown seaweed *C. cervicornis* [6] have demonstrated a progressive loss of mitochondrial membrane potential and cell death in *Leishmania amazonensis*. Desoti *et al.* [40] showed that the action of the elatol on *Trypanosoma cruzi* also evolve an oxidative stress, the commitment of mitochondrial function and ROS generation. The loss of mitochondrial membrane potential in DME-treated parasites may result in an increasing of ROS that, in higher amounts, have deleterious effects on parasites leading to cell death [33].

To better evaluate the physiologic effects of DME on the parasite membrane we used propidium iodide (PI), a membrane impermeant fluorescent dye, which is excluded from cells with intact plasma membranes, but binds to DNA and RNA in cells whose the membrane integrity was lost [41,42].

**Figure 7:**
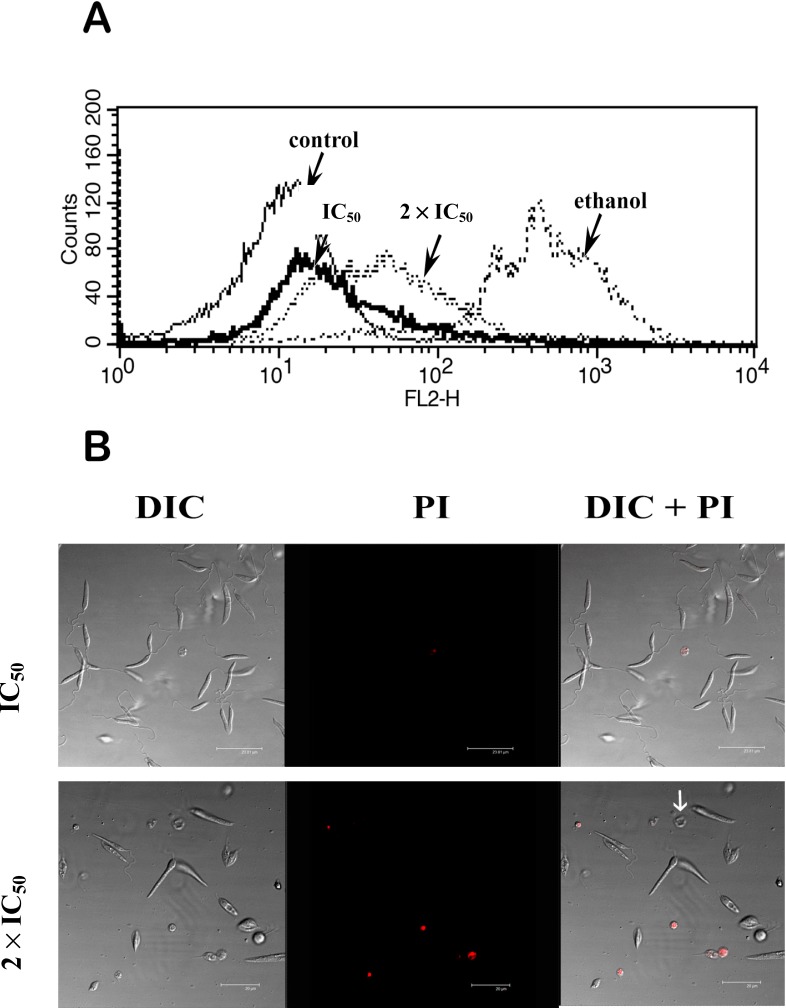
The effects of DME treatment on the promastigote plasma membrane integrity (**A**) Overlay flow cytometry histograms of control and treated cells submitted to PI staining. A slight increase in PI fluorescence signal could be observed in cells treated with 2 × IC_50_ of DME, whereas strong PI labeling could be observed in ethanol-treated parasites. (**B**) Confocal images cells treated with 1 × IC_50_ and 2 × IC_50_ of DME and submitted to staining with PI. Note the presence of cells with severely altered morphology not stained with PI (white arrow). DIC = Differential Interference Contrast.

Treatment of promastigotes with DME IC_50_ had no drastic change on the PI fluorescence intensity compared to the control cells, as observed by flow cytometry (Figure 7A) with only 5.91% of the cells showing positive for PI.

Conversely, the treatment of the promastigotes with ethanol, used for permeabilization of the plasma membrane, caused an intense shift in the PI fluorescence and 91.4% of the cells were labeled with PI. A discrete shift in red fluorescence could be observed in the 2 × IC_50_-treated cells, with about 15% of the cell population positive for PI. 

The confocal images showed that most untreated cells exhibited no labeling for PI, as well as normal elongated morphology (data not shown). No considerable changes in this profile were observed in the cells treated with the IC_50_. However, a slight increase in PI labeled cells could be detected in cells treated with the 2 × IC_50_ of DME. Dramatic morphological changes compatible with the loss of cell viability were present in all PI cells. Interestingly, some of the cells exhibiting severe morphological alterations were negative for this fluorescent marker. These results together with the data from cytometry suggest that necrosis is not the main mechanism of cell death elicited by DME. Actually, the ultrastructural analysis suggested an autophagic profile in DME-treated parasites with appearance of reticulum profiles surrounding organelles and concentric membranous structures in the cytoplasm. However, disorganization in inner nuclear structure, indicative of apoptosis was also observed in DME-treated parasites [35].

Autophagy represents a homeostatic cellular mechanism for the turnover of organelles and a degradation pathway, mainly in response to starvation, allowing the cell recycling molecules necessary for their survival. However, the autophagy may share common signal transduction elements with apoptosis besides inducing necrosis-like death [43]. The involvement of autophagy and apoptosis as the main mechanisms of DME-induced cell death, without substantial involvement of necrosis, represents an advantage, since both processes kill the parasites silently without triggering a host inflammatory response as observed in necrotic cells [35].

## 3. Experimental Section

### 3.1. Seaweed Collection and Extract Preparation

The individuals of seaweed *D. mertensii* were collected by free diving, at depths between 1–2 m in Itapuama Beach (8°17'S; 34°57'W), south cost of Pernambuco State, Brazil. The taxonomic identification was performed at the Museum of Oceanography of the Federal University of Pernambuco, where a voucher specimen was deposited under number MOUFPE 000.001. The specimens were cleaned in tap water, to remove sediments and epiphytes. The seaweed samples (20 g) were air-dried, milled to a powder and extracted at room temperature with a dichloromethane/methanol mixture (2:1). The crude extract of *D. mertensii* (DME) was evaporated at 40 °C using a rotatory vacuum evaporator. For anti-parasitic assays, the DME was initially diluted in 100% DMSO at the concentration of 50 mg/mL. Following, this solution was diluted in the culture to obtain a stock solution at 1 mg/mL. The stock solution was diluted again in the culture medium at different concentrations of DME. The concentration of DMSO never exceeded the concentration of 0.5%, which is non-toxic to the protozoa or the mammalian cells.

### 3.2. Parasites

Promastigote forms of *Leishmania amazonensis* (MHOM/77BR/LTB0016) were maintained in Schneider’s medium supplemented with 20% bovine foetal serum, at 26 °C and harvested during the exponential phase of growth. Amastigote forms were obtained from *L. amazonensis*-infected BALB/c peritoneal macrophages.

### 3.3. In Vitro Effects of DME on Promastigote Growth

*L. amazonensis* promastigote forms (1 × 10^6^ cells/mL.) were incubated at 26 °C in Schneider’s Drosophila medium supplemented with 10% FBS in the absence or presence of the different concentrations of DME (15.6–250 µg/mL). The cell density was determined daily using a Neubauer counting chamber. The drug concentration that inhibited culture growth by 50% (IC_50_) was estimated after 72 h of drug incubation by regression analysis using the SPSS 8.0 software. All experiments were performed in triplicate in two independent experiments.

### 3.4. In Vitro Effect of DME on Intracellular Amastigote Forms

Peritoneal macrophages from Balb/c mice were harvested and plated at 3 × 10^5^ cells/mL in a 24-well plate, containing RPMI medium supplemented with 15% inactivated fetal bovine serum. The macrophages were allowed to adhere for 24 h at 37 °C in 5% CO_2_ and then infected with promastigotes using a ratio 1:10 at 37 °C for 3 h. Non-interiorized parasites were removed by washing and the infected culture were incubated for 18 h in RPMI 1640 medium or treated with different concentrations of DME (18–144 μg/mL). The cultures were stained using the Panótico staining kit (Laborclin, Pinhais, Brazil.) The percentage of infected macrophages was determined by counting 100 randomly chosen cells in triplicate. The survival index was determined by multiplying the percentage of infected macrophages by the mean number of parasites per infected cell.

### 3.5. Cytotoxicity Assay

Peritoneal macrophages from Balb/c mice were plated at 6 × 10^5^ cells/well in 96-well plate containing 100 μL of RPMI medium, supplemented with 10% inactivated FBS, and incubated for 24 h at 37 °C in 5% CO_2_. After this time, the adhered macrophages were cultivated for 48 h in RPMI in the absence or presence of the different concentrations of DME (15.6 to 250 μg/mL). Treated and untreated cells were washed and incubated in fresh RPMI culture medium, containing 5 mg/mL of 3-(4,5-dimethylthiazol-2-yl)-2,5-diphenyltetrazolium bromide (MTT, Sigma-Aldrich, St. Louis, MO, USA), for an additional 3 h at 37 °C. After the incubation, the cells were solubilized in DMSO (100 μL/well) and the formazan precipitate derived from MTT reduction was determined spectrophotometrically at 540 nm. The 50% cytotoxic concentration (CC_50_) was determined by regression analysis using the software SPSS 8.0 for Windows. The selectivity index (SeI) was determined as the ratio of CC_50_ for the macrophages to IC_50_ for the protozoa. Each assay was carried out in quadriplicate in two independent experiments.

### 3.6. Nitric Oxide Production

In order to quantify the concentration of nitrite, 100 μL of supernatant of macrophages cultures treated or not with DME for 48 h were incubated with 100 μL of Griess reagent (1% sulphanilamide and 0.1% of *N*-(1-naphthyl)-ethylenediamine dihydrochloride/2.5% H_3_PO_4_ ) at room temperature for 10 min. The absorbance was measured in 540 nm at ELISA reader Benchmark Plus (Bio-Rad Laboratories, Philadelphia, PA, USA). The concentration of nitrite was determined using a standard curve of sodium nitrite.

### 3.7. Ultrastructural Assay

Promastigote forms of *L. amazonensis* treated or not with IC_50_ and 2 × IC_50_ for 48 h were harvested by centrifugation at 1500× *g*, washed in 0.1 M phosphate buffer, pH 7.2, and fixed for 2 h at 4 °C in a solution containing 2.5% glutaraldehyde/4% paraformaldehyde in 0.1 M phosphate buffer, pH 7.2. After washing in the same buffer, the cells were post-fixed for 1 h with 1% osmium tetroxide/0.8% potassium ferricyanide/5 mM CaCl_2_ in 0.1 M cacodylate buffer, pH 7.2. The cells were dehydrated in graded acetone series and embedded for 72 h at 60 °C in PolyBed 812 resin (PolySciences, Warrington, PA, USA). Ultrathin sections were stained with 5% uranyl acetate and lead citrate and observed in a Zeiss EM109 transmission electron microscope (TEM). For scanning electron microscopy (SEM), treated and untreated promastigotes were fixed and post-fixed as described above and allowed to adhere to poly-lysine-coated coverslips. The samples were dehydrated in ethanol, critical-point-dried with CO_2_, coated with a 20 nm-thick gold layer, and observed with a JEOL T-200 scanning electron microscope.

### 3.8. Flow Cytometry and Confocal Microscopy

For the analysis of the putative effects of the DME on the parasite plasma membrane and the mitochondrial membrane potential the fluorescent probes propidium iodide and rhodamine 123 were used, respectively. Promastigote forms of *L. amazonensis* (1 × 10^6^ cells/mL) were treated with IC_50_ and 2 × IC_50_ for 72 h. After treatment the parasites were incubated with 30 μg/mL propidium iodide or 10 μg/mL of rhodamine 123 for 15 min. Cells treated with ethanol or methanol were used as controls for propidium iodide and rhodamine 123 respectively. Afterward, the samples were analyzed in the FACSCalibur (Becton & Dickinson, San Jose, CA, USA) cytometer, using the Cell Quest software, in the channel FL2-H for PI and FL1-H for rhodamine 123. A total of 10,000 events were analyzed. Changes in the fluorescent intensity of rhodamine 123 were quantified using the variation index (VI) obtained by the equation (MT-MC)/MC, where MC is the mean of fluorescent intensity of control and MT the mean of treated cells. Negative values of VI correspond to membrane depolarization of mitochondrion [25]. Alternativelly, treated and non-treated parasites submitted to PI and Rho 123 were placed in 35 cm^3^ Mattek culture plates (Mattek Co., Ashland, MA, USA) and observed by confocal microscopy Leica SP2 AOBS (Leica, HM). The images were acquired using the lasers HeNe (488 nm) for PI and KrAg (543 nm) for rhodamine 123.

### 3.9. Ethical Standards

All experiments involving the use of experimental animals were performed in accordance to the ethical standards of Fundação Oswaldo Cruz and were approved by the ethics committee (CEUA-FIOCRUZ L-0001/08).

## 4. Conclusions

Our results showed that DME strongly inhibited the growth and cell viability of promastigote and amastigote forms of *Leishmania amazonensis* leading to cell death independent of necrosis but dependent on mitochondria. Although further studies on the chemical characterization and isolation of active compounds from DME are still needed, these results, together with the low toxicity presented by DME on mammalian cells, open new perspectives on the development of chemotherapeutic agents more effective against cutaneous leishmaniasis. Moreover, this work also shows the importance of bioprospecting studies highlighting the relevance of the Brazilian marine biodiversity as sources of potential natural compounds with pharmacological properties or biotechnological potential that could be used in the development of new drugs. The active extract DME deserves special attention in further studies to chemically characterize the bioactive compounds.

## References

[1] Barbosa J.P., Pereira R.C., Abrantes J.L., Cirne dos Santos C.C., Rabello M.A., Frugulhetti I.C., Teixeira V.L. (2004). *In vitro* antiviral diterpenes from the Brazilian brown alga *Dictyota pfaffii*. Planta Med..

[2] Sagar S., Kaur M., Minneman K.P. (2010). Antiviral lead compounds from marine sponges. Mar. Drugs.

[3] Kim S.K., Karadeniz F. (2011). Anti-HIV activity of extracts and compounds from marine algae. Adv. Food Nutr. Res..

[4] Genovese G., Tedone L., Hamann M.T., Morabito M. (2009). The mediterranean red alga Asparagopsi: A source of compounds against *Leishmania*. Mar. Drugs.

[5] Dos Santos A.O., Veiga-Santos P., Ueda-Nakamura T., dias Filho B.P., Sudatti D.B., Bianco E.M., Pereira R.C., Nakamura C.V. (2010). Effect of elatol, isolated from red seaweed *Laurencia dendroidea*, on *Leishmania amazonensis*. Mar. Drugs.

[6] Dos Santos A.O., Britta E.A., Bianco E.M., Ueda-Nakamura T., Filho B.P., Pereira R.C., Nakamura C.V. (2011). 4-Acetoxydolastane diterpene from the Brazilian brown alga *Canistrocarpus cervicornis* as antileishmanial agent. Mar. Drugs.

[7] Veiga-Santos P., Rocha K.J.P., Santos A.O., Ueda-Nakamura T., dias Filho B.P., Lautenschlager S.O.S., Sudatti D.B., Bianco E.M., Pereira R.C., Nakamura C.V. (2010). *In vitro* anti-trypanosomal activity of elatol isolated from red seaweed *Laurencia dendroidea*. Parasitology.

[8] Jones A.J., Grkovic T., Sykes M.L., Avery V.M. (2013). Trypanocidal activity of marine natural products. Mar. Drugs.

[9] Mancini I., Defant A., Guella G. (2007). Recent synthesis of marine natural products with antibacterial activities. Anti-Infect. Agents Med. Chem..

[10] Zubia M., Robledo D., Freile-Pelegrin Y. (2007). Antioxidant activities in tropical marine macroalgae from the Yucatan Peninsula, México. J. Appl. Phycol..

[11] Wattanadilok R., Sawangwong P., Rodrigues C., Cidade H., Pinto M., Pinto E., Silva A., Kijjoa A. (2007). Antifungal activity evaluation of the constituents of *Haliclona baeri* and *Haliclona cymaeformis*, collected from the Gulf of Thailand. Mar. Drugs.

[12] Friedman M.A., Fleming L.E., Fernandez M., Bienfang P., Schrank K., Dickey R., Bottein M., Backer L., Ayyar R., Weisman R. (2008). Ciguatera fish poisoning: Treatment, prevention and management. Mar. Drugs.

[13] Wang D. (2008). Neurotoxins from marine dinoflagellates: A brief review. Mar. Drugs.

[14] Rinehart K.L., Gloer J.B., Hughes R.G., Renis H.E., Mcgovren J.P., Swynenberg E.B., Stringfellow D.A., Kuentzel S.L., Li L.H. (1981). Didemnins: Antiviral and antitumor depsipeptides from a Caribbean tunicate. Science.

[15] Simmons T.L., Andrianasolo E., Flatt K.M.P., Gerwick W.H. (2005). Marine natural products as anticancer drugs. Mol. Cancer Ther..

[16] Bhakuni D.S., Rawat D.S. (2005). Bioactive Marine Natural Products.

[17] Maschek J.A, Baker B.J. , Amsler C.D. (2008). The chemistry of algal secondary metabolism. Algal Chemical Ecology.

[18] Costa-Lotufo L.V., Wilke D.V., Jimenez P.C., Epifanio R.A. (2009). Organismos marinhos como fonte de novos fármacos: Histórico & perspectivas. Quím. Nova.

[19] Molinski T.F., Dalisay D.S., Lievens S.L., Saludes J.P. (2009). Drug development from marine natural products. Nat. Rev. Drug Discov..

[20] Bianco E.M., de Oliveira S.Q., Rigotto C., Tonini M.L., da Rosa Guimarães T., Bittencourt F., Gouvêa L.P., Aresi C., de Almeida M.T., Moritz M.I. (2013). Anti-infective potential of marinhe invertebrates and seaweeds form the brazilian coast. Molecules.

[21] Harhay M.O., Olliaro P.L., Costa D.L., Costa C.H. (2011). Urban parasitology: Visceral leishmaniasis in Brazil. Trends Parasitol..

[22] WHO Neglected tropical diseases. http://www.who.int/neglected_diseases/en/.

[23] Markle W., Makhoul K. (2004). Cutaneous leishmaniasis: Recognition and treatment. Am. Fam. Physician.

[24] Monge-Maillo B., López-Velez R. (2013). Therapeutic options for visceral leishmaniasis. Drugs.

[25] Moura L.A., Almeida A.C.M., Domingos T.F., Ortiz-Ramirez F., Cavalcanti D.N., Teixeira V.L., Fuly A.L. (2014). Antiplatelet and anticoagulant effects of diterpenes isolated from the marine alga, *Dictyota menstrualis*. Mar. Drugs.

[26] Caamal-Fuentes E., Moo-Puc R., Freile-Pelegrín Y., Robledo D. (2014). Cytotoxic and antiproliferative constituentes from *Dictyota ciliolata*, *Padina sancate-crucis* and *Turbinaria tricostata*. Pharm. Biol..

[27] Vallim M.A., Pereira R.C., de Paula J.C., Teixeira V.L. (2005). The diterpenes from Dictyotacean marine brown algae in the Tropical Atlantic America region. Biochem. System. Ecol..

[28] Bianco E.M., Teixeira V.L., Pereira R.C., de Souza A.M., Nucci P., Afonso I.F., Rodrigues C.R., Castro H.C. (2009). Brown seaweed defensive chemicals: A structure-activity relationship approach for the marine environment. Nat. Prod. Commun..

[29] Bhargava P., Singh R. (2012). Developments in diagnosis and antileishmanial drugs. Interdiscip. Perspect. Infect. Dis..

[30] Kima P.E. (2007). The amastigote forms of Leishmania are experts at exploiting host cell processes to establish infection and persist. Int. J. Parasitol..

[31] Van Assche T., Deschacht M., da Luz R.A., Maes L., Cos P. (2011). Leishmania-macrophage interactions insights into the redox biology. Free Radic. Biol. Med..

[32] Fidalgo L.M., Gille L. (2011). Mitochondria and trypanosomatids: Target and drugs. Pharm. Res..

[33] Menna-Barreto R.F., de Castro S.L. (2014). The double-edged sword in pathogenic trypanosomatids: The pivotal role of mitochondria in oxidative stress and bioenergetics. Biomed. Res. Int..

[34] Silva C.F., Meuser M.B., de Souza E.M., Meirelles M.N.L., Stephens C.E., Som P., Boykin D.W., Soeiro M.N.C. (2007). Cellular effects of reversed amidines on *Trypanosoma cruzi*. Antimicrob. Agents Chemother..

[35] Menna-Barreto R.F., Gonçalves R.L.S., Costa E.M., Silva R.S.F., Pinto A.V., Oliveira M.F., de Castro S.L. (2009). The effects on *Trypanosoma cruzi* of novel synthetic naphthoquinones are mediated by mitochondrial dysfunction. Free Radic. Biol. Med..

[36] Vannier-Santos M.A., de Castro S.L. (2009). Electron microscopy in antiparasitic chemotherapy: A (close) view to a kill. Curr. Drug Targets.

[37] Shang X.J., Yao G., Ge J.P., Sun Y., Teng W.H., Huang Y.F. (2009). Procyanidin induces apoptosis and necrosis of prostate cancer cell line PC-3 in a mitochondrion-dependent manner. J. Androl..

[38] Fonseca-Silva F., Inacio J.D.F., Canto-Cavalheiro M.M., Almeida-Amaral E.E. (2011). Reactive oxygen species production and mitochondrial dysfunction contribute to quercetin induced death in Leishmania amazonensis. PLoS One.

[39] Forbes-Hernández T.Y., Giampieri F., Gasparrini M., Mazzoni L., Quiles J.L., Alvarez-Suarez J.M., Battino M. (2014). The effects of bioactive compounds from plant foods on mitochondrial function: A focus on apoptotic mechanism. Food Chem. Toxicol..

[40] Desoti V.C., Lazarin-Bidóia D., Sudatti D.B., Pereira R.C., Alonso A., Ueda-Nakamura T., dias Filho B.P., Nakamura C.V., Silva S.O. (2012). Trypanocidal action of (−)-elatol involves an oxidative stress triggered by mitochondia dysfunction. Mar. Drugs.

[41] Krysko D.V., Berghe T.V., D’Herde K., Vandenabeele P. (2008). Apoptosis and necrosis: Detection, descrimination and phagocytosis. Methods.

[42] Sandes J.M., Fontes A., Regis-da-Silva C.G., de Castro M.C., Lima-Junior C.G., Silva F.P., Vasconcellos M.L., Figueiredo R.C. (2014). Trypanosoma cruzi cell death induced by the Morita-Baylis-Hillman adduct 3-Hydroxy-2-methylene-3-(4-nitrophenylpropanenitrile). PLoS One.

[43] Ryter S.W., Mizumura K., Choi A.M.K. (2014). The impact of autophagy on cell death modalities. Int. J. Cell Biol..

